# Animal Models of Phage Therapy

**DOI:** 10.3389/fmicb.2021.631794

**Published:** 2021-01-28

**Authors:** Samuel Penziner, Robert T. Schooley, David T. Pride

**Affiliations:** ^1^Department of Medicine, University of California, San Diego, San Diego, CA, United States; ^2^Department of Pathology, University of California, San Diego, San Diego, CA, United States

**Keywords:** phage therapy, phage, bacteriophage, animal models, bacterial infections

## Abstract

Amidst the rising tide of antibiotic resistance, phage therapy holds promise as an alternative to antibiotics. Most well-designed studies on phage therapy exist in animal models. In order to progress to human clinical trials, it is important to understand what these models have accomplished and determine how to improve upon them. Here we provide a review of the animal models of phage therapy in Western literature and outline what can be learned from them in order to bring phage therapy closer to becoming a feasible alternative to antibiotics in clinical practice.

## Introduction

The discovery of bacteriophages, viruses that infect bacteria, can be traced to the early 1900’s when Frederick Twort and Félix d’Hérelle each observed unexplained clearings on agar plates of bacteria. Both men found that the clearings were transmissible, but it was d’Hérelle who postulated that they were caused by viruses and coined the term “bacteriophages,” meaning “eaters of bacteria” (Chanishvili, 2012). D’Hérelle also saw the potential of bacteriophages (or “phages”) as therapy against bacterial diseases. In 1919 he used them to cure a 12-year-old boy of Shigella dysentery, and eventually phage therapy was adopted around the world for the treatment of skin infections, diarrheal illnesses, and even the bubonic plague (Stone, 2002; Chanishvili, 2012; Myelnikov, 2018). However, following the development of antibiotics and concerns about inconsistent results, phage therapy waned in Western medicine in the 1940’s (Eaton and Bayne-Jones, 1934; Chanishvili, 2012; Myelnikov, 2018). Countries in Eastern Europe continued to implement phage therapy in medical practice, though these studies were not conducted in such a way that would meet Western regulatory or pharmaceutical approval (Merril et al., 2003).

Antibiotics have served as the bedrock for the treatment of bacterial diseases and yet, due to the growing crisis of antibiotic resistance, routine infections are becoming difficult to treat (Lesho and Laguio-Vila, 2019). According to some estimates, by 2050 ten million people a year will die from multidrug resistant (MDR) bacterial infections (World Bank Group, 2017). Despite the critical need for new antibiotics, the antibiotic pipeline is not expected to keep pace with the rate of resistance (Årdal et al., 2020). This has led to a renewed interest in phage therapy. Phage therapy offers unique advantages over antibiotics such as the narrow specificity of each individual bacteriophage (allowing for preservation of the body’s endogenous bacterial flora), ability to increase in number after administration (by replicating within the bacterial host), and capability to penetrate biofilms (Viertel et al., 2014; Rehman et al., 2019).

At present, phage therapy in humans has consisted of compassionate use cases (Schooley et al., 2017; Chan et al., 2018; Law et al., 2019; McCallin et al., 2019) and a few clinical trials hampered by small sample sizes or methodological issues (Wright et al., 2009; Sarker et al., 2016; Jault et al., 2019). The bulk of data on phage therapy lies in animal studies (Table 1). While these studies cannot replace human trials, they can impart valuable information about working with different infection models, bacterial species, phages, dosing strategies, and endpoints. They also provide data on the pharmacokinetics (absorption, metabolism, distribution, and elimination throughout the body), immunogenicity (interaction with the immune system), and safety of bacteriophages *in vivo*. This review describes how animal models of phage therapy have been constructed and what can be learned from their results in order to guide further work in the field.

**TABLE 1:** Studies on phage therapy^a^ according to organ system, bacteria, and animal model.

System	Bacteria	Animal Model	References
CNS	*Escherichia coli*	Rats	Pouillot et al., 2012
GI	*Escherichia coli*	Calves	Smith and Huggins, 1983; Smith et al., 1987
GI	*Escherichia coli*	Mice	Galtier et al., 2016
GI	*Escherichia coli*	Pigs	Smith and Huggins, 1983; Jamalludeen et al., 2009
GI	*Escherichia coli*	Sheep	Smith and Huggins, 1983; Raya et al., 2006
GI	*Salmonella enterica*	Chicken	Colom et al., 2015; Tie et al., 2018
GI	*Salmonella enterica*	Mice	Dallal et al., 2019
GI	*Salmonella enterica*	Quails	Ahmadi et al., 2016
GI	*Vibrio cholerae*	Mice	Jaiswal et al., 2014
GI	*Vibrio cholerae*	Rabbits	Bhandare et al., 2019
GI	*Vibrio parahaemolyticus*	Mice	Jun et al., 2014
Keratitis	*Pseudomonas aeruginosa*	Mice	Furusawa et al., 2016
Osteomyelitis	*Staphylococcus aureus*	Rabbits	Kishor et al., 2016
Otitis	*Pseudomonas aeruginosa*	Dogs	Hawkins et al., 2010
PNA	*Acinetobacter baumannii*	Mice	Jeon et al., 2016, 2019; Hua et al., 2017
PNA	*Burkholderia cenocepacia*	Mice	Carmody et al., 2010; Semler et al., 2014
PNA	*Burkholderia pseudomallei*	Mice	Guang-Han et al., 2016
PNA	*Escherichia coli*	Mice	Dufour et al., 2015, 2016, 2019
PNA	*Klebsiella pneumoniae*	Mice	Chhibber et al., 2008; Singla et al., 2015; Anand et al., 2020
PNA	*Pseudomonas aeruginosa*	Mice	Debarbieux et al., 2010; Morello et al., 2011; Alemayehu et al., 2012; Henry et al., 2013; Yang et al., 2015; Pabary et al., 2016; Roach et al., 2017; Waters et al., 2017; Chang et al., 2018; Forti et al., 2018; Abd El-Aziz et al., 2019
PNA	*Staphylococcus aureus*	Rats	Prazak et al., 2019
PNA	*Staphylococcus aureus*	Mice	Takemura-Uchiyama et al., 2014
Sinusitis	*Staphylococcus aureus*	Sheep	Drilling et al., 2014
SSTI	*Acinetobacter baumannii*	Mice	Regeimbal et al., 2016
SSTI	*Acinetobacter baumannii*	Pigs	Mendes et al., 2013
SSTI	*Acinetobacter baumannii*	Rats	Mendes et al., 2013; Shivaswamy et al., 2015
SSTI	*Klebsiella pneumoniae*	Mice	Kumari et al., 2010; Chadha et al., 2016
SSTI	*Mycobacterium ulcerans*	Mice	Trigo et al., 2013
SSTI	*Pseudomonas aeruginosa*	Rats	Mendes et al., 2013
SSTI	*Pseudomonas aeruginosa*	Pigs	Mendes et al., 2013
SSTI	*Staphylococcus aureus*	Mice	Capparelli et al., 2007; Chhibber et al., 2013
SSTI	*Staphylococcus aureus*	Rabbits	Wills et al., 2005
SSTI	*Staphylococcus aureus*	Rats	Mendes et al., 2013; Chhibber et al., 2017
SSTI	*Staphylococcus aureus*	Pigs	Mendes et al., 2013
Systemic	*Acinetobacter baumannii*	Galleria mellonella	Regeimbal et al., 2016; Jeon et al., 2019
Systemic	*Acinetobacter baumannii*	Mice	Leshkasheli et al., 2019
Systemic	*Burkholderia cenocepacia*	Galleria mellonella	Seed and Dennis, 2009
Systemic	*Citrobacter freundii*	Mice	Kaabi and Musafer, 2019
Systemic	*Clostridioides difficile*	Galleria mellonella	Nale et al., 2016a
Systemic	*Enterobacter cloacae*	Galleria mellonella	Manohar et al., 2018
Systemic	*Enterococcus faecalis*	Zebrafish	Al-Zubidi et al., 2019
Systemic	*Enterococcus faecalis*	Mice	Uchiyama et al., 2008
Systemic	*Enterococcus faecium*	Mice	Biswas et al., 2002
Systemic	*Escherichia coli*	Galleria mellonella	Manohar et al., 2018
Systemic	*Escherichia coli*	Mice	Smith and Huggins, 1982; Wang et al., 2006b; Pouillot et al., 2012; Dufour et al., 2016; Schneider et al., 2018; Kaabi and Musafer, 2019
Systemic	*Escherichia coli*	Quails	Naghizadeh et al., 2019
Systemic	*Haemophilus influenzae*	Mice	Kaabi and Musafer, 2019
Systemic	*Klebsiella pneumoniae*	Galleria mellonella	Manohar et al., 2018
Systemic	*Klebsiella pneumoniae*	Mice	Hung et al., 2011; Kaabi and Musafer, 2019
Systemic	*Moraxella catarrhalis*	Mice	Kaabi and Musafer, 2019
Systemic	*Pasteurella multocida*	Mice	Chen et al., 2019
Systemic	*Pseudomonas aeruginosa*	*Drosophila melanogaster*	Heo et al., 2009
Systemic	*Pseudomonas aeruginosa*	Galleria mellonella	Forti et al., 2018
Systemic	*Pseudomonas aeruginosa*	Mice	Wang et al., 2006a; Watanabe et al., 2007; Heo et al., 2009; Shivshetty et al., 2014; Kaabi and Musafer, 2019
Systemic	*Pseudomonas aeruginosa*	Zebrafish	Cafora et al., 2019
Systemic	*Salmonella enterica*	Caenorhabditis elegans	Tang et al., 2019
Systemic	*Salmonella enterica*	Mice	Capparelli et al., 2010; Tang et al., 2019
Systemic	*Staphylococcus aureus*	Mice	Capparelli et al., 2007; Takemura-Uchiyama et al., 2014; Oduor et al., 2016
Systemic	*Vibrio parahaemolyticus*	Mice	Jun et al., 2014
Systemic	*Vibrio vulnificus*	Mice	Cerveny et al., 2002
UTI	*Escherichia coli*	Mice	Dufour et al., 2016

*CNS, central nervous system; GI, gastrointestinal; PNA, pneumonia; SSTI, skin and soft tissue infection; UTI, urinary tract infection. ^a^Phage therapy refers to the administration of the first phage dose after the administration of bacteria (as opposed to phage prophylaxis which entails the first phage dose at or before the administration of bacteria). All of the studies listed include phage therapy as at least one component of their experiments conducted.*

## Bacteria and Phages Employed in Animal Models

Since phages amount to the most abundant biological entity on earth with a population estimated at >10^30 (Hendrix, 2002), they infect many species of bacteria and can be studied in a variety of bacterial models. Bacteria used in animal studies tend to be those relevant in clinical practice, particularly with predilection for antibiotic resistance. Many studies have focused on the ESKAPE pathogens (*Enterococcus faecium*, *Staphylococcus aureus*, *Klebsiella pneumoniae*, *Acinetobacter baumannii*, *Pseudomonas aeruginosa*, and *Enterobacter cloacae*) (Debarbieux et al., 2010; Kumari et al., 2010; Chhibber et al., 2013; Jeon et al., 2016), a group of organisms designated by the World Health Organization as serious threats to global health based on high rates of multidrug resistance (Tacconelli et al., 2017). Animal studies have also used phage therapy to treat organisms such as *Escherichia coli* (Schneider et al., 2018), *Salmonella enterica* (Dallal et al., 2019), *Vibrio cholerae* (Bhandare et al., 2019), *Mycobacterium ulcerans* (Trigo et al., 2013), and *Burkholderia pseudomallei* (Guang-Han et al., 2016). Meanwhile, the relationship between antibiotic resistance and phage resistance (or sensitivity) in these organisms is a complex one. For example, while Chan et al. (2016) demonstrated an instance in which MDR *P. aeruginosa* exhibited a trade-off between antibiotic resistance and phage resistance through alterations in an efflux pump, work by Burmeister et al. (2020) provided an example where phage-resistant *E. coli* mutants exhibited increased resistance to tetracycline via changes to the bacterial lipopolysaccharide.

Phages kill bacteria in a process referred to as the lytic cycle where they adsorb to the bacterial surface, eject genetic material into the host, use bacterial replication machinery to generate phage progeny (and other proteins), and lyse the cell to release the newly generated virions (Gordillo Altamirano and Barr, 2019). Virulent phages are those which can only infect bacteria through the lytic cycle, whereas temperate phages can perform either the lytic cycle or an alternate process, the lysogenic cycle, in which phage DNA is integrated into the host’s. Temperate phages are often considered unsuitable for phage therapy because lysogeny enables the transfer of genetic material (such as virulence factors or antibiotic resistance genes) to and between bacteria (Górski et al., 2018). As such, usually virulent phages are sought for use in phage therapy. Phages can be isolated from environments where the host bacteria can be found, such as sewage, soil, and river water (Smith and Huggins, 1982; Wang et al., 2006a). Alternatively, libraries of previously identified phages can be screened against the bacterial isolate of interest. *In vitro* phage-susceptibility testing is then performed with spot assays on agar plates (Chhibber et al., 2008; Heo et al., 2009) or with growth curves in liquid media which provide dynamic data on killing activity (Soothill, 1992; Tanji et al., 2005; Wang et al., 2006b). DNA sequencing provides phage characterization and is also essential to identify genes involved in lysogeny, such as integrases, which would implicate a phage as temperate. Unfortunately the functions of many phage genes remain unknown (Hatfull and Hendrix, 2011), and screening out phages based on putative gene functions does not always identify temperate phages.

The majority of virulent bacteriophages that have been discovered belong to the taxonomic order *Caudovirales*. *Caudovirales* are non-enveloped, tailed bacteriophages with icosahedral or elongated heads and genomes comprised of double-stranded DNA (Ackermann, 1998). Traditionally the order *Caudovirales* has been divided into three families—*Myoviridae*, *Siphoviridae*, and *Podoviridae*, though other families within the order have been discovered recently (Walker et al., 2019). *Siphoviridae* previously appeared to be the most abundant of the *Caudovirales*, but this varies from bacterial host to host (Ackermann, 1998). At present, there is insufficient evidence to indicate that one family of *Caudovirales* is more effective at killing bacteria than another.

## Constructing the Infection Model and Administering Phage Therapy

In the animal model of phage therapy, “infection” refers to the administration of bacteria. “Therapy” or “treatment” refers to the administration of phages *after* administration of bacteria and is distinct from phage “prophylaxis” in which phages are given *at or before* bacterial inoculation. To establish infection, the bacterial isolate is administered to the animal via the appropriate route (e.g., intranasally for pneumonia) at a known dose designated in colony-forming units (CFU) (Shivshetty et al., 2014). Investigators often set an endpoint (such as 100% animal fatality at 48 h) in order to determine the appropriate bacterial dose to employ in the subsequent phage therapy experiment (Biswas et al., 2002; Roach et al., 2017). This often requires trialing various doses before arriving at the desired infectious endpoint (Yang et al., 2015).

Bacteriophages require multiple steps of preparation before administration. Animals such as mice can only tolerate the administration of small volumes of liquid, especially when administered intravenously or intranasally; phages must therefore be concentrated to accommodate the necessary inoculum (Guang-Han et al., 2016; Roach et al., 2017). Inoculum size is dictated by the desired multiplicity of infection (MOI), which is the ratio of viral plaque forming units (PFU) to bacterial CFU. For example, if the animal receives 10^8 CFU of bacteria and an MOI of 100 is needed, then the volume that the animal receives must contain 10^10 PFU of bacteriophages. Once the bacteriophages have been concentrated, they are then purified further, often via ultracentrifugation using cesium chloride (Biswas et al., 2002) followed by removal of the solvent with dialysis. Experiments usually take the additional step of removing endotoxin (Roach et al., 2017), a lipopolysaccharide released from the cell wall of Gram negative bacteria that can be extremely toxic. For phage therapy in human subjects, all of these steps are crucial to ensure purity, uniformity, and safety of bacteriophages prior to administration.

To initiate the phage therapy experiment, animals receive the bacterial isolate at the previously established dose. At a later time point the intervention group receives bacteriophages, and the control group receives buffer without phages, heat-inactivated phages (Wang et al., 2006a), or no treatment. Many proof-of-concept experiments have focused on phage monotherapy (Wang et al., 2006b; Capparelli et al., 2010; Carmody et al., 2010; Yang et al., 2015); however, since it is well established that phage-resistant bacterial variants emerge during treatment (Oechslin, 2018), studies have also examined phage cocktails, which contain mixtures of different bacteriophages (Forti et al., 2018; Seo et al., 2018; Naghizadeh et al., 2019). Cocktails provide a broader range of bacterial killing and may be able to mitigate the emergence of resistance by targeting different host receptors. For this reason, phage cocktails are used whenever possible in compassionate-use cases in humans.

The route of phage administration can be oral (Bhandare et al., 2019), intravenous (Capparelli et al., 2010), intraperitoneal (Biswas et al., 2002), subcutaneous (Capparelli et al., 2010), intramuscular (Naghizadeh et al., 2019), intranasal (Alemayehu et al., 2012), intratracheal (Chang et al., 2018), or topical (Mendes et al., 2013). For some models of infection, one route of phage administration fails even if adequate in other models or with other bacterial pathogens. For example, intravenous and intraperitoneal phages have not cured any animal models of *P. aeruginosa* pneumonia; only intranasal and intratracheal phages have worked (Debarbieux et al., 2010; Roach et al., 2017; Chang et al., 2018). Experiments may examine various MOIs of bacteriophages necessary to cure infection, which frequently lie between 1 and 1000 (Biswas et al., 2002; Jeon et al., 2016). Unsurprisingly, higher MOIs are more effective than lower MOIs at killing bacteria (Morello et al., 2011), though it is likely no further benefit exists after reaching a certain threshold.

A few points bear consideration on the limitations of experimental design and how animal work differs from clinical practice. First, *in vivo* animal experiments have the luxury of using bacterial isolates for which virulent phages have already been discovered. In human patients who require phage therapy, the bacterial isolate is obtained without *a priori* knowledge of any susceptibility to phages in a laboratory’s phage library. In the critically ill, where delays in treatment are associated with decreased survival (Kumar et al., 2006), physicians cannot wait for isolate testing before initiating therapy. Phage cocktails, which could offer broad host-range empirically, may be a means of using phages when isolate susceptibility is not yet known. Models that use phage cocktails, such as those by Forti et al. (2018), Naghizadeh et al. (2019), and Prazak et al. (2019) warrant further attention.

Second, unlike in animal experiments in which the bacterial inoculum is known, human infections are caused by an amount of bacteria that often cannot be quantified, and applying accurate phage dosing by MOI in humans is difficult. It is also possible that the bacterial burden in a human infection could exceed the amount of bacteria used in any animal experiment by orders of magnitude; if attempting to mimic the MOIs of animal studies, this would call for larger doses of phages than implemented in any of these experiments. This still may be feasible based on the size of humans compared to mice, though interestingly, a number of case reports in humans have used phage inocula of comparable size to those used in animals. For example, in Schooley et al. (2017) treatment of a disseminated *A. baumannii* infection, intravenous phage doses each contained 5 × 10^9 PFU of virus, while Law et al. (2019) used phage doses of 4 × 10^9 PFU to successfully treat *P. aeruginosa* in a cystic fibrosis patient. In both cases, many doses of phages were given over a period of weeks, which may have compensated for the size of the individual doses. Nevertheless, it remains quite possible that high MOIs in humans may be unnecessary based on the self-amplifying nature of bacteriophages.

Finally, infections in animal studies compared to human infections also differ by their rates of progression. In most animal studies, experimentally induced infections progress rapidly, often killing animals within 48–72 h in the absence of phage treatment (Debarbieux et al., 2010; Roach et al., 2017; Schneider et al., 2018). As a result, phage therapy to treat these animal infections needs to be given immediately or soon after bacterial inoculation. Since human infections rarely progress as quickly as the infections in these animal models (e.g., a patient with pneumonia may wait a few days before even presenting to the hospital), phage therapy in humans will likely be administered after a delayed amount of time. Significant delays in treatment limit efficacy in animal models, though a theoretical benefit to a delay would be that phages could replicate to higher titers in the presence of a high bacterial burden. A few animal studies have managed to recreate delayed conditions of human infections, notably the chronic *P. aeruginosa* pneumonia model established by Waters et al. (2017) and the non-lethal *S. aureus* bacteremia model constructed by Capparelli et al. (2007). More indolent infection models like these are needed, particularly of pneumonia and systemic infections, that allow animals to survive through at least 5 or 7 days without treatment and still can be cured when phage therapy is given after a delayed amount of time. Until then, it will remain unclear whether phages can reliably cure infections unless given soon after their onset.

## Efficacy of Phage Therapy in Animal Models

Research into phage therapy in the West laid relatively dormant from the 1940’s onward but reappeared again in the 1980’s with animal work conducted by Smith and Huggins (1982, 1983) and Smith et al. (1987). In one of their first experiments, they infected mice intramuscularly or intracerebrally with the bacterium *E. coli.* A single intramuscular dose of one phage with activity against the host bacteria reduced mortality more than multiple doses of various antibiotics (Smith and Huggins, 1982). A year later, they found that certain phages could treat *E. coli* diarrhea in calves, pigs, and lambs. They also found that phage-treated calves that survived *E. coli* infection continued to excrete phages in their feces, at least until the quantity of the pathogenic *E. coli* strain excreted was low (Smith and Huggins, 1983). Subsequent work demonstrated that phage cocktails could also be used in place of individual phages (Smith et al., 1987). Soothill was the next to examine phages in animals, albeit as prophylaxis rather than treatment. In their 1992 study (Soothill, 1992), phages prevented intraperitoneal *A. baumannii* and *P. aeruginosa* infections. Another study showed phages could prevent skin graft infections in guinea pigs if administered prior to the introduction of *P. aeruginosa* (Soothill, 1994).

While these studies were informative, there was little discussion in the early 1990’s about the need for phage therapy in humans, so animal studies were not pursued robustly. However, as multidrug antibacterial resistance developed into a global crisis (Murray, 1994; Tenover and McGowan, 1996), the prospects of phage therapy attracted more attention (Alisky et al., 1998; Stone, 2002), prompting a new wave of animal studies.

### Systemic Infections

Systemic infections are those that result in bacteremia and/or dissemination to multiple organs. The 1992 experiment by Soothill (1992) was a model of systemic infection but, as mentioned, it focused on prophylaxis. Cerveny et al. (2002) injected mice subcutaneously with *Vibrio vulnificus* to induce bacteremia and then administered phages intravenously. In this study, phages only had an effect when injected simultaneously as prophylaxis, whereas when given at 6 or 12 h after bacterial inoculation, they provided no mortality benefit. However, in the same year, Biswas et al. (2002) were able to successfully treat a systemic infection induced by intraperitoneal injections of vancomycin-resistant *E. faecium* in mice. Phages administered after 45 min at an MOI of 0.3 or 3.0 rescued 100% of the animals; when delayed until 5 h at an MOI of 3, 100% of mice still survived. Even with delays in treatment of 18 and 24 h, at which point all mice were quite ill, approximately 50% of the animals survived and recovered completely.

Most subsequent work has examined infections with *P. aeruginosa* and *E. coli*, though other studied bacterial species have included *S. aureus*, *A. baumannii, K. pneumoniae*, *S. enterica*, *Pasteurella multocida*, and *Vibrio parahaemolyticus* (Capparelli et al., 2007; Capparelli et al., 2010; Hung et al., 2011; Jun et al., 2014; Oduor et al., 2016; Chen et al., 2019; Leshkasheli et al., 2019). Studies often show that efficacy depends on the timing of administration. For example, in a study by Wang et al. (2006a) on imipenem-resistant *P. aeruginosa* infections, phages were 100% effective when administered 1 h after infection at an MOI of 200, but cure rates dropped to 50 and 20%, respectively, when treatment was delayed to 3 and 6 h. The same group studied drug-resistant *E. coli* infections and noted that all mice died when intraperitoneal phage treatment was delayed to 6 h (Wang et al., 2006b). Capparelli et al. (2007), however, showed that treatment could be quite delayed in the context of a more indolent infection; the investigators administered a low dose of 5 × 10^6 CFU of *S. aureus* intravenously and then at day 10 administered either no treatment or phages at an MOI of 1000. At day 20, phage-treated mice had sterilized their spleens, kidneys, hearts, and blood, whereas those tissues remain infected in the control mice.

The most common routes of phage administration for systemic infections entail intraperitoneal (Wang et al., 2006a; Uchiyama et al., 2008), intravenous (Oduor et al., 2016), oral (Jun et al., 2014), or intramuscular (Heo et al., 2009) dosing. For treatment of *P. aeruginosa*, Watanabe et al. (2007) determined that intravenous and intraperitoneal methods were superior to oral, and Heo et al. (2009) demonstrated that intraperitoneal was superior to intramuscular. In humans, phages for systemic infections will likely require intravenous or oral administration since these are the routes via which medications are typically administered in medical practice. More studies are needed to examine these routes of therapy.

### Pneumonia

Pneumonia models of phage therapy initially lagged behind other models of infection but have since taken a prominent role in the literature. Chhibber et al. (2008) used *K. pneumoniae* at a concentration of 10^8 CFU/ml to induce pneumonia in mice and simultaneously gave them phages intraperitoneally. As with the early systemic infection models (Soothill, 1992; Cerveny et al., 2002), phages successfully provided prophylaxis against infection but could not treat it. Nevertheless, this study helped set the stage for the other pneumonia models of phage therapy to come.

Studies have frequently focused on *P. aeruginosa*, a common culprit of hospital-acquired pneumonia and cystic fibrosis exacerbations. Additionally, mortality associated with MDR *P. aeruginosa* in ventilator-associated pneumonia exceeds 35% (Ramírez-Estrada et al., 2016). Debarbieux et al. (2010) demonstrated that pneumonia responded to phage treatment, but phage administration was intranasal, and timing of administration was crucial. 100% of mice survived in the group treated with phages 2 h after bacterial inoculation, and 75% of mice survived when phages were given after 4 or 6 h. The study also examined various MOIs required to treat infection and found that an MOI of at least 10 was necessary. Unsurprisingly, other studies have found that higher MOIs are superior to lower ones (Morello et al., 2011). Administering phages early is also superior to waiting, even if by only an hour (Yang et al., 2015). There are two studies that capture efficacy of delayed treatment. Waters et al. (2017) established a chronic form of lung infection using a *P. aeruginosa* strain found in patients with cystic fibrosis. The study successfully cleared infection in mice with a phage at an MOI of 10 that was given as late as 48 h after bacterial inoculation. Abd El-Aziz et al. (2019) also treated pneumonia successfully at 12 h after infection using an MOI of 0.1. Only 60% of the control group developed severe disease, underscoring how interpretations of phage efficacy are dependent on the severity of the underlying bacterial infection.

In addition to treating *P. aeruginosa*, phages have shown efficacy in bacterial pneumonias caused by *S. aureus* (Prazak et al., 2019), *E. coli* (Dufour et al., 2016), *A. baumannii* (Jeon et al., 2016), *K. pneumoniae* (Anand et al., 2020), *B. pseudomallei* (Guang-Han et al., 2016), and *Burkholderia cenocepacia* (Carmody et al., 2010), all of which can cause MDR pneumonia in critically ill patients or those with cystic fibrosis. Dufour et al. (2015) found that bacteriophage treatment enabled 100% survival of mice infected with a highly virulent *E. coli* strain. A study of *A. baumannii* pneumonia observed that administration of phages at an MOI of 10 given 30 min after infection resulted in 100% survival (Jeon et al., 2016). In a *B. pseudomallei* study, phages in magnitude of 10^8 PFU rescued 1/3rd of the mice, which was superior to outcomes in control mice that were not given phages (Guang-Han et al., 2016).

Since some pathogens do not lead to rapidly progressive pneumonia, particularly those that form biofilms in the airways of patients with cystic fibrosis, investigators have also evaluated responses to phage therapy using quantitative microbial endpoints rather than simply assessing improvement in illness severity or mortality. Debarbieux, Roach, Forti, and others (Debarbieux et al., 2010; Henry et al., 2013; Dufour et al., 2015; Roach et al., 2017) infected mice with bioluminescent bacteria and used IVIS spectroscopy to display the decrease in luminescence that resulted from phage treatment. Pabary et al. (2016) administered phages 24 h after *P. aeruginosa* infection and then performed bronchoalveolar lavages (BALs) on mice at 48 h. There was complete clearance of bacteria in 6 of 7 phage-treated mice, and the median CFU/ml was significantly lower compared to controls that were not given phages. Another study found no bacteria detected in the lungs after lung extraction at 72 h (Abd El-Aziz et al., 2019), and the use of aerosolized phage by Chang et al. (2018) was able to decrease bacterial loads in lung homogenates at both 4 and 24 h compared to controls.

Synergy between the immune system and bacteriophages likely plays an important role in killing bacteria and has only partially been elucidated. Roach et al. (2017) showed that neutrophils are an essential part of controlling both phage-sensitive and emergent phage-resistant bacterial variants as a means of ensuring effective treatment. Work by Abd El-Aziz et al. (2019) showed that the addition of phages to a mixture of human serum and bacteria enhanced serum killing activity. Multiple studies provide evidence that the immune system generates antibodies against bacteriophages (Biswas et al., 2002; Wang et al., 2006a; Shivshetty et al., 2014), though this likely would impair rather than bolster activity against bacterial infection.

As with systemic models of infection, the efficacy of phage therapy is highly dependent on experimental conditions. Again, models are needed to prove phage efficacy when administered more than a few hours after infection.

### Diarrheal Infections

Enteritis (infection of the small bowel) and colitis (infection of the large bowel) are common models of infection studied for the use of phage therapy. Because diarrheal infections primarily affect the lumen of the gastrointestinal tract, these studies allow for experimentation with oral phages (without requiring their systemic absorption), simplifying the administration of phage therapy.

Many studies have focused on *E. coli*, one of the most common causes of diarrheal illness in humans throughout the world (Leung et al., 2019). As mentioned earlier, Smith and Huggins (1983) looked at treatment of *E. coli* diarrhea in calves, pigs, and lambs. Raya et al. (2006) infected 8 sheep orally with *E. coli* strain O157:H7. Four of the sheep were given an oral phage at an MOI of 10, and 2 days later all eight sheep were euthanized. Bacterial counts in the colons of treated sheep were 2–3 log_10_ lower than the untreated ones. Jamalludeen et al. (2009) challenged pigs orally with 10^10 CFU of Enterotoxigenic *E. coli* (ETEC) and 24 h later administered a combination of two phages at a total of 10^8 PFU for three doses, separated 6 h apart. Compared to controls that did not receive phages, oral phage administration reduced the development of diarrhea and quantity of ETEC in pig feces.

Since *V. cholera* infects 3–5 million people annually and causes 21,000–143,000 deaths a year, studies have explored management with bacteriophages (Jaiswal et al., 2014; Yen et al., 2017; Bhandare et al., 2019). In a study by Jaiswal et al. (2014), mice received oral *V. cholerae* and then daily dosing of a phage cocktail (at an MOI of 0.5), the antibiotic ciprofloxacin, or oral rehydration. Phage cocktails reduced the burden of *V. cholerae* in tissue and blood by 3 log_10_ compared to rehydration, which provided no benefit, though ciprofloxacin produced superior results in reducing *V. cholerae* by 4 log_10_. Despite the inferiority of phages to ciprofloxacin in this study, the specificity of phages could preserve endogenous host flora compared to the broad host-range of antibiotics. In a cholera study in rabbits, animals were infected orally and then administered oral phages 6 h later. Eleven of 17 infected control rabbits developed symptoms of enteritis, whereas none of the nineteen phage-treated animals showed signs of disease. Phage treatment also significantly reduced the amount of *V. cholerae* recovered from the intestines of treated rabbits compared with untreated controls (Bhandare et al., 2019).

Other studies on infections of the lower gastrointestinal tract have examined enteric pathogens such as *S. enterica* and *V. parahaemolyticus*. A study of *S. enterica* serovar enteritidis in mice found that oral phages administered at an MOI of 10 1 day after gavage with the bacteria showed no signs of infection, and no bacteria were isolated from the liver; mice not administered phages all died at the end of the experiment and exhibited clear liver pathology associated with recovered Salmonella isolates (Dallal et al., 2019). In a model of *V. parahaemolyticus* enteritis, mortality rates were twice as high in control mice compared to mice treated intraperitoneally with phages (Jun et al., 2014).

While many studies focus on phages as prophylaxis, it is important to note that due to the unpredictability of infections in humans, some animal models of prophylaxis lack relevance to phage therapy in humans. An exception to this lies in the prophylaxis of bacterial diarrheal illnesses, which are widespread in developing countries due to contamination of water sources and are caused by a more limited range of bacterial species. In Eastern European literature, one of the largest human phage therapy studies conducted in the 1960’s indicated that prophylactic tablets of oral phages could reduce the acquisition of Shigella dysentery (Chanishvili, 2012). In animal work on *V. cholera*, Yen et al. (2017) demonstrated that a cocktail of three phages reduced bacterial colonization of mice, optimally when given 6 h prior to bacterial inoculation, as opposed to 12 or 24 h prior. Apart from their experiment on cholera treatment, Bhandare et al. (2019) studied prophylaxis by giving rabbits oral phages at 10^9 PFU 6 h prior to infecting them with 5 × 10^8 CFU of bacteria; none of the rabbits developed diarrhea. Meanwhile, Nale et al. (2016b) studied the prevention of *Clostridioides difficile*, the most common culprit of hospital-acquired diarrhea. Hamsters received inoculation with *C. difficile* spores and a cocktail of phages simultaneously and every 8 h thereafter at an MOI of 10,000. At 36 h, phages reduced *C. difficile* bacterial counts in the GI lumen by at least 4 log_10_ and delayed mortality by greater than a day. Of note, this study used temperate phages rather than strictly lytic ones.

### Other Infections

There are a number of models of skin and soft tissue infections. Wills et al. (2005) injected *S. aureus* subcutaneously into the flanks of rabbits. Phages administered subcutaneously simultaneously as prophylaxis prevented abscesses but when administered as treatment at 6, 12, or 24 h after infection, they did not. Kumari et al. (2010) examined murine wounds that were infected with *K. pneumoniae*. A single intraperitoneal injection of phages could rescue 73% of the animals when delayed to 6 h after burn/bacterial challenge. Delay in treatment resulted in lower survival, but even with delays of 12 and 18 h, at which point all the mice were ill, 46 and 26% of the animals, respectively, could be rescued and went on to recover completely. In a model of *S. aureus* foot infections in diabetic mice, phages were more effective than the antibiotic linezolid, though the two agents together showed superior efficacy (Chhibber et al., 2013). Shivaswamy et al. (2015) used topical phage treatments for infected wounds in both mice and pigs; phages were able to reduce bacterial counts caused by *P. aeruginosa* and *S. aureus*. Trigo et al. (2013) studied a slower form of skin/soft tissue infection caused by the bacteria *M. ulcerans*, waiting until 33 days post-infection to administer a single dose of bacteriophages. At day 68, footpads of non-treated mice started showing signs of ulceration, while in mycobacteriophage-treated mice the progression of swelling halted after day 91 post-infection.

Other models of infection include orthopedic, urinary tract, and CNS infections. Kishor et al. (2016) induced methicillin resistant *S. aureus* (MRSA) osteomyelitis of the distal femur in rabbits. Mice given phages in three doses every 2 days beginning in the 3rd week of infection were compared to phage treatment started in the 6th week of infection. All mice in the former group were cured based on microbiologic, radiologic, and histopathologic examinations. Those given phages starting in the 6th week showed some radiologic features of chronic osteomyelitis, but wounds healed, and the sites became sterile. Dufour et al. (2016) injected *E. coli* into the bladder of mice and the following day administered bacteriophage treatment intraperitoneally at an MOI of 200. At 48 h, bacterial loads in the kidneys decreased by 2 log_10_ compared to controls that received no phages. To treat a CNS infection in mice caused by ESBL *E. coli*, Pouillot et al. (2012) administered intraperitoneal phages 1 h later at a dose of 10^8 PFU. All animals survived to day 5. Phage concentration in the CSF was 10-fold higher than that in blood, demonstrating the capacity of phages to cross the blood-CSF barrier; work in humans by Ghose et al. (2019) has also recently confirmed this finding. Other studies have shown effective treatment of keratitis in mice (Furusawa et al., 2016), otitis media in dogs (Hawkins et al., 2010), sinusitis in sheep (Drilling et al., 2014), and systemic infections in zebrafish (Al-Zubidi et al., 2019; Cafora et al., 2019) and moth larvae (Hall et al., 2012; Augustine et al., 2014; Nale et al., 2016a; Manohar et al., 2018; Jeon et al., 2019), supporting the idea that phage therapy can be studied in a broad range of infection models and animal species.

The application of phage lysins (rather than the phages themselves) to fight infections is also an area of intense investigation. Ectolysins are structural enzymes on the phage virion that facilitate phage entry into bacterial cells, while endolysins are non-structural enzymes responsible for the release of phages after they have replicated within the cytoplasm (Kim et al., 2019). In both cases, lysins degrade the peptidoglycan of the bacterial cell wall. Endolysins have been found to kill bacteria effectively when they are applied extrinsically. Lysins have been studied most extensively with Gram positive bacteria where they can easily access the cell wall peptidoglycan; however, lysins active against Gram negative bacteria have also been found (Lood et al., 2015; Raz et al., 2019), and lysin-bacteriocin fusion molecules have been developed that can translocate the outer membrane of Gram negative organisms (Heselpoth et al., 2019), a technique that would allow lysins to gain access to the otherwise poorly accessible peptidoglycan protected by the Gram negative outer cell membrane. As with models of phage therapy, lysins have shown efficacy in a variety of animal models of infection (Daniel et al., 2010; Vouillamoz et al., 2013; Schuch et al., 2014; Díez-Martínez et al., 2015; Lood et al., 2015; Raz et al., 2019), and a phase 3 clinical trial in humans studying a lysin for the treatment of *S. aureus* bacteremia is currently underway (Globe Newswire, 2020).

## Phage Pharmacokinetics

The pharmacokinetics of bacteriophages are more complex than traditional antibiotics because they replicate after infecting their bacterial hosts. Thus, their behavior also differs depending on whether a bacterial host is present or absent, and the persistence of phages *in vivo* may suggest that infection has not been eradicated. Methods of characterizing phage kinetics have relied on serial blood and stool measurements (Cerveny et al., 2002; Seo et al., 2018), bronchoalveolar lavages (Morello et al., 2011), homogenizing organs after animal euthanasia at various time points (Chhibber et al., 2008; Takemura-Uchiyama et al., 2014), and in rare instances, phage labeling (Rusckowski et al., 2004, 2008).

Phages have been detected in all major organs following phage therapy, including the lungs, spleen, liver, kidney, stomach, and intestines (Carmody et al., 2010; Takemura-Uchiyama et al., 2014; Schneider et al., 2018). They have also been detected in brain tissue, indicating that they can pass through the blood-brain barrier (Pouillot et al., 2012; Ghose et al., 2019). When Pouillot et al. (2012) and Ghose et al. (2019) injected phages intraperitoneally, they found higher concentrations of phages in the spleen and kidney than compared to blood and concluded that this represented phage trapping by these organs.

Even in the absence of a host bacterial infection, phages persist in most body compartments for 2–3 days. Cerveny et al. (2002) initially estimated the half-life of phages in the blood of uninfected mice to be 2.2 h. In uninfected mice receiving intraperitoneal phage injections, Chhibber et al. (2008) found that maximum phage concentrations in blood, peritoneal fluid, and lungs occurred at 6 h and were negligible by 36 h. Jun et al. (2014) also found peak concentrations in blood occurred at 6 h following intraperitoneal injection, whereas after oral administration peak levels occurred at 12 h. Via both routes, no phages were detected at 48 h. Yen, Kumari, and others have arrived at similar results (Kumari et al., 2010; Yen et al., 2017). These data support the Uchiyama et al. (2009) two-compartment pharmacokinetic model where phages initially distribute to organs and then are slowly eliminated from the body. Interaction with the tissues of individual organ systems also likely contributes to these dynamics and is extremely complex. An example would be the subdiffusive movement of phages in mucous secretions which Barr et al. (2015) showed serves to increase the interactions between phages and bacteria.

Route of administration influences phage concentrations in various organs. For example, phage concentrations seem to achieve higher levels throughout the body when injected intramuscularly compared to intraperitoneally (Heo et al., 2009). Lungs contain higher concentrations following inhalational therapy compared to intraperitoneal therapy (Carmody et al., 2010). Subcutaneous administration results in lower and more delayed blood concentrations compared to intraperitoneal injection (Pouillot et al., 2012). Intravenous therapy functions similarly to intraperitoneal therapy, with the exception that the latter results in higher intraperitoneal phage concentrations.

In animals infected with bacteria that serve as hosts for bacteriophages, the persistence and concentration levels of phages also differ. When phages were administered in the presence of a systemic MRSA infection, phages could be detected for at least 96 h in the blood (Oduor et al., 2016). Watanabe et al. (2007) induced gut-derived sepsis with oral *P. aeruginosa*, which was followed by administration of oral phages. After 8 days, high concentrations of phages remained in the blood and liver when high levels of bacteria were still detected, demonstrating that phages continue to replicate as long the bacterial host remains present. Similarly, in an *E. coli* sepsis model in which IV phages were administered, they could be detected in the spleen up to 2 weeks after infection in the surviving mice (Schneider et al., 2018). Meanwhile, Takemura-Uchiyama et al. (2014) compared phage concentrations in mice with *S. aureus* sepsis and those that were not infected but given phages alone. Findings conveyed that phage concentrations in the blood, lung, and liver were 10^7, 10^5, and 10^3 times higher in the infected mice. In the Debarbieux et al. (2010) model of *P. aeruginosa* pneumonia, infected mice treated with phages demonstrated higher phage concentrations in their lungs by 1 log_10_ value compared to mice given only phages.

A couple of studies have labeled bacteriophages with technetium and administered them to infected animals (Rusckowski et al., 2004, 2008). These studies did not use phages intended to kill their bacterial host but instead focused on using the phages to locate the sites of the bacterial infection with imaging. These studies offer promise as an alternative means of assessing bacteriophage distribution within the body. Unfortunately, they only labeled parent phages (the directly administered phages) and were unable to characterize the distribution or behavior of progeny phages (phages produced after the parent phages replicated). Imaging techniques to detect both parent and progeny phages in phage therapy would offer valuable information moving forward.

## Toxicity and Immune Response

Phage therapy has proven safe in the many animal experiments thus far (Uchiyama et al., 2008; Shivshetty et al., 2014; Oduor et al., 2016). For example, Uchiyama et al. (2008) gave mice repeated intraperitoneal phage injections seven times a day every 4 days for 2 months and found this had no overt clinical effects. Chen et al. (2019) injected mice with 10^8 PFU of phages intraperitoneally and saw no abnormal histological changes in the main organs, and mice experienced no adverse health effects. Human studies have been equally reassuring (Sarker et al., 2016; Gindin et al., 2019; Ooi et al., 2019; Petrovic Fabijan et al., 2020).

Phages also do not induce significant amounts of inflammation. Dufour et al. (2019) found that phages could elicit cytokine production above baseline, but this was localized, phage dependent, and had no observable clinical consequences. Other work has shown that when uninfected mice were given inhalational or intraperitoneal phages, no appreciable levels of either TNF-α or MIP-2 were observed in the lungs (Carmody et al., 2010). In the study by Debarbieux et al. (2010), cytokine levels of IL-6 and TNF-α were as low in animals given phages as those given PBS solution. Roach et al. (2017) arrived at similar conclusions. These studies indicate that phage treatments alone do not result in significant inflammatory responses in the absence of host bacteria (Morello et al., 2011).

Nevertheless, phages do influence immune responses. Phages have served as antigenic stimuli in the evaluation of immunodeficiencies, and phage proteins have been used as vehicles for vaccine antigens (Górski et al., 2012). The immune response against phages results in their inactivation and clearance and has important implications in the efficacy of therapy. From an innate immunity standpoint, both the non-cellular complement system and phagocytosis lead to inactivation and clearance of phages from circulation (Da̧browska, 2019; Hodyra-Stefaniak et al., 2019). Phagocytosis in the liver and spleen occurs rapidly (Inchley, 1969; Geier et al., 1973) and is also upregulated in the presence of concomitant systemic inflammation (such as that produced by a bacterial infection) (Hodyra-Stefaniak et al., 2015). In comparison to the liver, the spleen retains intact phages for longer periods by non-destructively capturing antigens using Schweigger-Seidel capillary sheaths (collections of macrophages in splenic capillary walls). This process may allow the spleen to serve as an ongoing source of antigens to stimulate antibody production (Geier et al., 1973), and in this way phagocytosis serves as the initial step leading to an adaptive immune response (Da̧browska, 2019).

Adaptive immunity to phages is characterized by a strong humoral response. In one animal study, 1 dose of intraperitoneal phages caused anti-phage IgG titers to increase 26-fold, peaking at day 40 (Wang et al., 2006a). In another study, IgG titers rose 3,800-fold above baseline after phage administration (Biswas et al., 2002). In a study by Hodyra-Stefaniak et al. (2015), both IgM and IgG antibodies were able to neutralize phages rapidly in pre-immunized mice compared to those that were initially phage-naïve, and Huff et al. (2010) found that an increase in IgG titers from a prior phage exposure resulted in a 40% decrease in phage efficacy when treating colibacillosis in poultry. In a study using PEGylation to extend phage half-life, Kim et al. (2008) showed this was ineffective if given to animals already exposed to the same phage previously. This suggested that PEGylation could not prevent the adaptive immune response from rapidly eliminating the phage.

Interestingly, human studies have demonstrated low levels of phage-neutralizing antibodies in patient serum even prior to phage administration, likely due to pre-exposure from the ubiquity of phages in the environment (Górski et al., 2012). The clinical relevance of this remains to be determined, but it introduces the possibility that adaptive immunity could contribute to inactivation of phage therapy upon initial treatment administration. The rapid development of bacterial resistance is often pointed to as a source of potential phage treatment failure, but it is likely that the immune clearance and inactivation of phages will also play an important role.

## Conclusion

Global antibiotic resistance has created urgency for phage therapy as an alternative to antibiotics. Studies in animals should serve as a guide to applying phage therapy in human diseases. The animal studies reviewed here reveal that phage therapy can work in all models of infection and on many species of bacteria. They have also demonstrated its safety. Nevertheless, there is still much to learn. Standardization of regimens, optimization of pharmacokinetic modeling, and development of models that mimic the time course of infection in human diseases are needed. Moving forward, animal models of phage therapy will need to address these issues if we hope to bring phage therapy into routine clinical practice in the 21st century.

## Author Contributions

SP wrote the review. DP and RS edited it. All the authors contributed to the article and approved the submitted version.

## Conflict of Interest

The authors declare that the research was conducted in the absence of any commercial or financial relationships that could be construed as a potential conflict of interest.
